# Topical non‐steroidal anti‐inflammatory drugs are not the mainstay of prophylaxis and treatment for pseudophakic cystoid macular oedema

**DOI:** 10.1111/ceo.13591

**Published:** 2019-08-08

**Authors:** Lewis M. Levitz, Chris H. L. Lim, Andrzej Grzybowski, Stephen J. Kim

**Affiliations:** ^1^ Vision Eye Institute Melbourne Victoria Australia; ^2^ Department of Ophthalmology National University Hospital Singapore; ^3^ Department of Ophthalmology University of Warmia and Mazury Olsztyn Poland; ^4^ Institute for Research in Ophthalmology Foundation for Ophthalmology Development Poznan Poland; ^5^ Vanderbilt University School of Medicine Nashville Tennessee

1

We read with interest the review by Han et al^1^ However, we would like to highlight several aspects of the quoted literature which may be misconstrued.

The authors have stated in both their abstract and conclusion that “Topical NSAIDs [non‐steroidal anti‐inflammatory drugs] remain the mainstay of prophylaxis and treatment of PCMO [pseudophakic cystoid macular oedema].” This is not supported by the content of the review and has the potential to mislead its audience.

Han et al referenced a Cochrane systematic review which examined the role of NSAIDs as prophylaxis against PCMO.^2^ The authors graded the level of evidence as very low to low‐certainty and reported concerns surrounding biases in all reviewed studies identified in the existing literature. Lim et al have further cautioned that any possible efficacy demonstrated as part of this analysis may be exaggerated given the study designs employed.

A systematic review by Kessel et al was further cited to provide evidence to support the preferential use of NSAIDs compared to corticosteroids.^3^ Although the original article recommended that NSAIDs should be started before planned surgery and that the use of NSAIDs after cataract surgery may prevent inflammation and macular oedema, these recommendations were not supported by their review. Following a challenge, this was later clarified in a subsequent reply.^4^ Most significantly, the review did not factor in potential discrepancies between treatment arms of included studies, where topical NSAIDS were preferentially dosed and compared against lower potency topical corticosteroid formulations. For example, the majority of studies reviewed by Kessel et al used fluorometholone 0.1%, which has very poor penetration into the aqueous and is essentially a surface corticosteroid. This review therefore does not accurately reflect real‐life practice patterns.

In a similar fashion, the PREMED study quoted was unable to show a visual benefit in patients assigned to receive NSAIDs. This is not surprising since PCMO often resolves spontaneously and can be compatible in mild to moderate cases with good vision. Further, there is no standard means to diagnose PCMO and thus definitions and incidence vary widely among published studies. We agree with the authors' suggestion that confusion surrounding the use of NSAIDs may have arisen from the lack of an accepted diagnostic criteria for PCMO.

The article by Donnenfeld et al has also been included as evidence that PCMO was lower in groups where NSAIDs were commenced between 1 and 3 days preoperatively.^5^ However, evaluation for the presence of PCMO was performed at 2 weeks post‐operatively in this study, which may be early for the identification of PCMO, which typically peaks at 4 to 6 weeks post‐operatively. Furthermore, preoperative structural macular imaging was not performed in these patients to definitively exclude pre‐existing macular oedema. The original authors have also noted that differences were not significant on statistical testing. We feel that the clinical impact and significance of this reference is overstated and may mislead readers.

The review article does allude to the challenges faced in providing advice on prophylaxis of PCMO when citing the report by the American Academy of Ophthalmology. A subsequently published editorial by the same author however questioned how prescribing NSAIDs for routine cataract surgery had become popularized without compelling evidence of visual benefit to patients.^6^

The authors have provided a significant review of a complex topic. However, the statement that topical NSAIDs remain the mainstay in the prophylaxis and treatment of PCMO is not supported by the literature reviewed. We hope that this letter may raise awareness of these issues to the readership.

## CONFLICT OF INTEREST

None declared.
